# A Scan Statistic for Binary Outcome Based on Hypergeometric Probability Model, with an Application to Detecting Spatial Clusters of Japanese Encephalitis

**DOI:** 10.1371/journal.pone.0065419

**Published:** 2013-06-13

**Authors:** Xing Zhao, Xiao-Hua Zhou, Zijian Feng, Pengfei Guo, Hongyan He, Tao Zhang, Lei Duan, Xiaosong Li

**Affiliations:** 1 Department of Biostatistics, West China School of Public Health, Sichuan University, Chengdu, Sichuan, China; 2 Department of Biostatistics, School of Public Health and Community Medicine, University of Washington, Seattle, Washington, United States of America; 3 Office for Disease Control and Emergency Response, Chinese Center for Disease Control and Prevention (China CDC), Beijing, China; 4 School of Computer Science, Sichuan University, Chengdu, Sichuan, China; 5 State Key Laboratory of Software Engineering, Wuhan University, Wuhan, Hubei, China; Stony Brook University, Graduate Program in Public Health, United States of America

## Abstract

As a useful tool for geographical cluster detection of events, the spatial scan statistic is widely applied in many fields and plays an increasingly important role. The classic version of the spatial scan statistic for the binary outcome is developed by Kulldorff, based on the Bernoulli or the Poisson probability model. In this paper, we apply the Hypergeometric probability model to construct the likelihood function under the null hypothesis. Compared with existing methods, the likelihood function under the null hypothesis is an alternative and indirect method to identify the potential cluster, and the test statistic is the extreme value of the likelihood function. Similar with Kulldorff’s methods, we adopt Monte Carlo test for the test of significance. Both methods are applied for detecting spatial clusters of Japanese encephalitis in Sichuan province, China, in 2009, and the detected clusters are identical. Through a simulation to independent benchmark data, it is indicated that the test statistic based on the Hypergeometric model outweighs Kulldorff’s statistics for clusters of high population density or large size; otherwise Kulldorff’s statistics are superior.

## Introduction

In epidemiological studies, it is often important to evaluate whether the occurrence of a disease is randomly distributed or tends to occur as clusters over time and/or space after adjusting for known confounding factors, which may provide clues to the etiology of the disease [1]. In addition, outbreak detection becomes possible thanks to growing geographically referenced health-related data, such as data from sales of Over-the-Counter (OTC) Healthcare Products.

Likelihood ratio based spatial scan statistic is a cluster detection test. Because of its ability of both identifying localized clusters and evaluating their significance, the spatial scan statistic becomes more popular relative to other statistical methods for disease clustering. In order to investigate excessive risk of disease after adjusting for the unevenly distributed population, Kulldorff proposed the spatial scan statistic for the binary outcome in his seminal papers [2], [3]. Those included the Bernoulli and the Poisson models. The spatial scan statistic quickly has become a popular research field and various new methods have been developed, which could roughly be divided into two classes: those extending the shape of the scanning window to detect irregularly shaped clusters [4]–[11], and those modifying the test statistic to exploit more information [12]–[17] or to accommodate more complex data structures, such as multivariate data [18]–[20], ordinal data [21], survival data [22], [23], normally distributed data [24], [25] and multinomial data [26]. For all methods, the significance of the test statistic is evaluated using the Monte Carlo test [27].

While a great variety of methods have been developed for diverse purposes, the two classic models for the binary outcome play a central role in the spatial scan statistic due to their wide application. The Bernoulli model is more appropriate for case-control data, while the Poisson model is more appropriate for case-population data. When the number of cases is small compared to the target population, the two models approximate each other. Both of them are based on the likelihood ratio test theory and use the likelihood ratio (LR), a generalized measuring index of clustering for each window, thereby making windows of different size comparable. In a word, the larger the LR of a window is, the more likely it is a true cluster. The window with the largest LR is called the most likely cluster (MLC) and the test statistic is the largest LR. In general, as summarized by Kulldorff [28], a test for spatial randomness adjusted for an inhomogeneity comprises 7 steps, of which the 3rd step is to construct a measuring index for each defined area and the 5th step is to define a summary quantification for all defined areas. The LR and the largest LR correspond with the 3rd and 5th steps, repsectively. Neill [29] presents a heuristic interpretation that the LR is a sort of distance away from the null hypothesis of no clustering. To the best of our knowledge, quite on the contrary, the LR is an index of measuring the closeness to the alternative hypothesis of an existing window of clustering. However, it is interesting to study whether there exists an index of measuring the departure from the null hypothesis of no clustering and, if so, how the new measuring index performs compared with the existing methods.

In this paper, we apply the Hypergeometric probability model to construct a likelihood function under the null hypothesis, which sometimes is complementary to the alternative hypothesis. The idea originates from the email exchange with Dr. Kulldorff and another method from Dr.Wong et al for syndrome surveillance, which is named WSARE [30]. By analogy with our situation, WSARE employs the P value of testing heterogeneity of proportions inside and outside each window as a measure of the departure from the null hypothesis. That is, the window with the minimum P value is the ‘most strange window’ (in Dr.Wong’s words) and the minimum P value is the test statistic. The proposed and existing methods are both applied to the real data of Japanese Encephalitis (JE) in Sichuan province, China. A simulation between the proposed test statistic and the Kulldorff’s statistics is carried out using a set of independent benchmark data.

## Materials and Methods

### JE Data

In 2003, there was a outbreak of SARS in China, and this exposed the underdevelopment of the public health system in handling public health emergencies in China. The China Central government requested to strengthen the construction of an infectious disease and public health emergency system, with focus on promoting the timeliness, sensitivity and accuracy of reporting. The Chinese Center for Disease Control and Prevention (CCDC) made the construction of a new operation model, Chinese Information System for Infectious Diseases Control and Prevention (CISIDCP). CISIDCP was established on the basis of individual cases and public health emergencies. A Virtual Private Network (VPN) has been constructed using the information safety technology, and information of individual cases is directly reported to the national database through the internet. This system will report 39 notifiable infectious diseases to CCDC within 24 hours. However, the management is classified into national, provincial, prefecture and county levels. CISIDCP makes feedback with health authority departments at every level. In 2005, CISIDCP had covered at least 93.3% of medical units at the county and above.

JE is among the 39 notifiable infectious diseases and, therefore JE case will be reported routinely by CISIDCP. JE is a vector-borne viral disease with a high mortality rate and a high percentage of neuropsychiatric sequelae. The JE virus is spread by marsh birds and intensified by pigs, mainly transmitted via the bite of infected Culex mosquito. Humans are dead-end hosts [31]. Many of the ecological, environmental, climatic and human behavioral factors are involved in the spread of the JE virus [32]. Contextual determinants of JE include irrigated rice farming, pig rearing and the rural population. Sichuan is a province in Southwest China. It is one one of the major agricultural production bases of China, including rice and pork production. Hence, Sichuan province is a high-incidence region for JE with the incidence of JE ranked the 5th in 2009 among 31 provinces in Mainland China. As a subordinate unit of CCDC, Sichuan Center for Disease Control and Prevention (SCDC) has the permission to access the data of Sichuan from CISIDCP. It is interesting for SCDC to investigate the geographic distribution pattern of JE. This analysis may help further learn the disease cluster areas and influencing factors of JE, finally assisting health officials in allocating the health resources.

### Notation and Kulldorff’s Test Statistics for the Binary Outcome

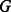
: the whole study region divided into many counties

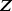
: a circular window in the study region

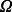
: all overlapped windows formed by circles of arbitrary radius 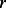 centered in each one of counties, 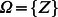

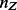
: the number of population within window 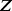

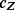
: the observed number of cases within window 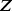

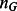
: the total population in the study region

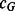
: total number of cases in the study region

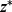
: one cluster such that all individuals within 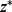 have probability 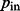 be a case and 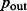 is the same probability for individuals outside 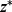

When scanning for the high rates only, such as identifying areas with high rate of leukemia [33] or Breast cancer [34], Kulldorff’s statement on the hypotheses is as following: 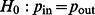, 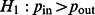 for 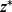. Under the null hypothesis, the expected number of cases within 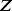, 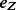, is calculated as follows:
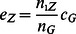
(1)


In the framework of likelihood ratio ratio test, the final test statistic for the Poisson model is the likelihood ratio maximized over 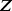:
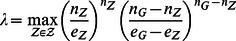
(2)where



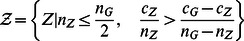
(3)The search space for the candidate cluster is ristricted by equation 3, where the first inequality specifies the maximum spatial cluster size, and the second inequality determines the aim of scanning for high risk area. Mathematical details have been provided by Kulldorff [3]. Sometimes it is interesting to identify areas with low rates only, such as detecting low clusters of sex ratio [35]. We just need to change the direction of the second inequality. The window 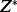 attaining the maximum is defined as the Most Likely Cluster (MLC). The MLC is least likely to be a chance occurrence under the null hypothesis. And the test statistic is the 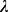 in equation 2. The significance of the test statistic is evaluated by Monte Carlo test.

### A Test Statistic based on the Hypergeometric Probability Model

As with the Poisson- and Bernoulli-based statistics, the Hypergeometric-based statistic has two aims: detecting the potential clusters; and evaluating the significance of the detected clusters.

#### Detecting

The null hypothesis signifies complete spatial randomness with each individual in the study region, implying that each person in the study region are equally likely to become the case. There are 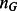 individuals of which 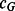 are cases in total. Under null hypothesis, we can think of that all 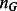 individuals are equally likely to be ‘labeled’. For a window 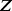 with 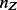 people, the probability of 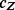 ‘labeled’ individuals has probability as:
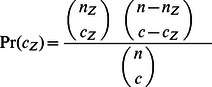
(4)


This is the classic application of Hypergeometric distribution, sampling from a finite population without replacement. The window 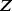 with the minimum probability is least likely occur under the null hypothesis. This can be written:
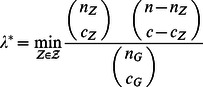
(5)


Where the 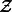 is the same as Kulldorff's method, which is defined by equation 3. The window 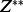 attaining the minimum probability is least likely occur under the null hypothesis. We may call this window the most strange window, as it have the minimum probability of satisfying the null hypothesis that 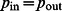.

#### Test of significance

The test statistic is the 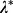 in equation 5. To evaluate whether the identified cluster is statistically significant or just can be explained by random noise, the 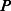 value is obtained through Monte Carlo test, by comparing the rank of the test statistic from the real data set with those from the random replications. The test statistic is calculated for each random replication as well as for the real data set, and if the latter is among the 5 percent lowest, then the test is significant at the 0.05 level. When there are multiple clusters in the data set, many methods can deal with this problem [3], [36], [37].

## Results

### Detection of Clusters of JE in Sichuan Province

We applied both the proposed and existing methods to analyze data on JE from 2009 in Sichuan province, China. Our analysis used both methods to investigate whether the high JE incidence is evenly spread over Sichuan province. This would examine whether any observed clusters of JE cases could be explained by chance alone, or whether there were clusters of statistical significance.

**Figure 1:**
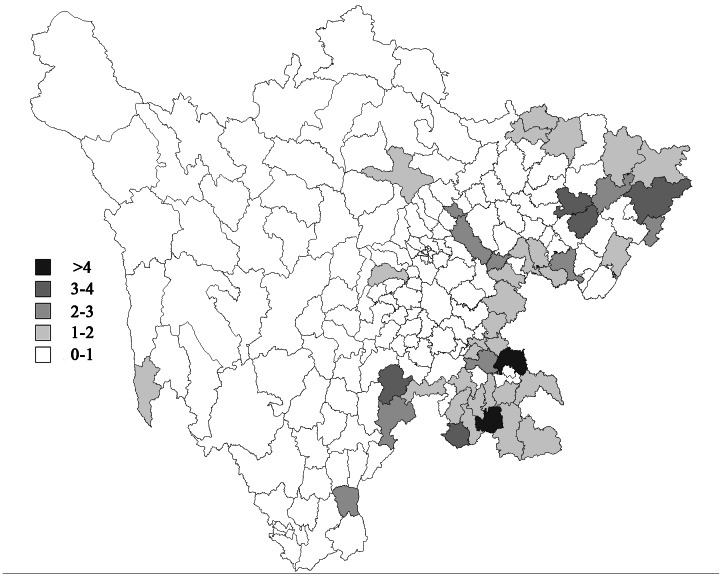
Choropleth map of empirical Bayes estimates of relative risk of Japanese encephalitis in Sichuan province in 2009.

In 2009, Sichuan province had a population of 81,379,919 and consisted of 181 counties. It had a total number of 598 cases, in which 2 cases lost the geographical information. The JE cases data in Sichuan province came from CISIDCP and this data was not publicly available. The population data came from the Nation Bureau of Statistics of China (http://www.stats.gov.cn/tjsj/ndsj/). To eliminate the random noise in the incidence map, we utilized a method of empirical Bayes estimate to visualize the spatial distribution of JE in Sichuan (Figure 1). It seems that there is a high risk in the east borderline region, especially in the northeast and southeast areas.

**Figure 2:**
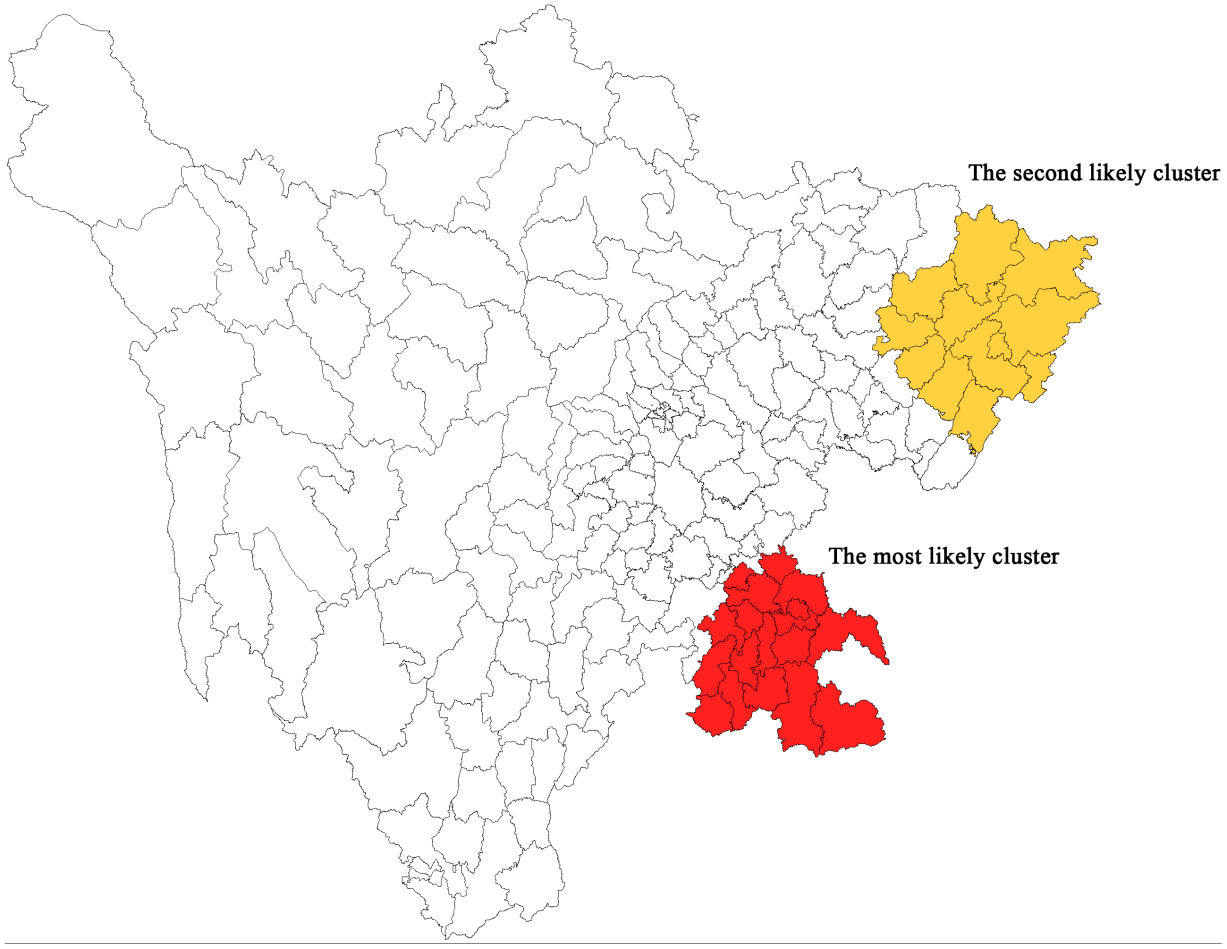
Detected clusters of Japanese encephalitis in Sichuan province in 2009. On a significance level of 0.05, the test statistics based on Poisson and Hypergeometric models obtained almost the same results. They detected two significant clusters, the most likely cluster in the southeast with 18 counties and one secondary cluster in the northeast with 12 counties, both with P value of 0.0001. They differs slightly on statistically insignificant clusters.

Because of the case-population data structure, we used the Poisson model representing Kulldorff’s method. The 9,999 random data were generated under the null hypothesis to evaluate the significance of the detected clusters. On a significance level of 0.05, the two methods obtained almost the same results (Figure 2). They detected two significant clusters, the most likely cluster in the southeast with 18 counties and one secondary cluster in the northeast with 12 counties. Both clusters had a P value of 0.0001. In addtion, the two methods detected identical areas as a third likely cluster, but with different P values: 0.0909 for the existing method and 0.1334 for the new method. This area is not colored in the figure. Overall, the detected clusters account for 53.2% (317/596) of the JE cases in Sichuan province. Cao [38] had analyzed the influencing factors of JE in the southwest of China on the Prefecture-city level, an administrative division below a province and above a county in China’s administrative structure, but found no statistically significant factors. As Cao presented, this is probably due to the little spatial variation of the influencing factors in the southwest of China. On the other hand, the financial support may, on a large extent, determine the spatial variation of JE in Sichuan. We ranked the counties by GDP per capita in a descending order. It turned out that 86.7% (26/30) of the counties in the significant clusters ranked in the last two thirds of the 181 counties, and 33.3% (10/30) ranked in the last one third parts. As pointed by Zhen [39], the less-developed region of Sichuan province was often short of funds for JE control, especially the remote rural areas. These constitute the high risk areas in Sichuan, which indicates that more financial and policy support is required to control JE in these areas.

### Simulation Study

#### Benchmark data

To carry out the comparison study between the two kinds of methods, we use public domain benchmark data sets. The benchmark data sets are based on the 1990 female population in the 245 counties and county equivalents in the Northeastern United States, consisting of the states of Maine, New Hampshire, Vermont, Massachusetts, Rhode Island, Connecticut, New York, New Jersey, Pennsylvania, Delaware, Maryland and the District of Columbia. Each county is represented by a centroid coordinate. The benchmark data was created by Kulldorff, M., T. Tango and P.J. Park (2003) to investigate the performance of different statistical methods for clustering. They have been used in many research studies [40]–[43]. It is available at ‘http://www.satscan.org/datasets.html’. The benchmark data and how it was generated has been described in detail elsewhere [40]. We provide a brief summary here.

Under the null hypothesis of no clustering, 99,999 random data sets were generated by randomly allocating 600 cases to the various counties, with probabilities proportional to the county population. The null data is used to estimate the critical values, which is the cut-off point for the significance. Hot-spot clusters were generated by setting the relative risk in some counties to be larger than 1. Different real hot-spot clusters corresponds to different combinations of population density, number of counties. For each kind of hot-spot clusters, 10,000 random data sets were generated using a multinomial probability distribution with the relative risks such that if the exact location of the real cluster was known in advance, the power to detect it should be 0.999. In order to clearly examine the performance of these methods when applied in high and low population density, we focused on the relatively extreme combinations. urban area, rural area, rural & mixed area and urban & mixed area.

#### Evaluation criteria

The sensitivity (SEN) and the positive predictive value (PPV) were estimated to examine the performance of different methods. The SEN and PPV of the spatial scan statistic were introduced by Huang et al [22], and can be defined in terms of either the number of regions or the population. First, we define the SEN as the probability of detecting the regions that actually constitute the cluster, i.e, proportion of the number of regions correctly detected from the true clusters.
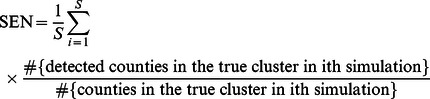
(6)


Where 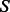 is the total number of simulations. The PPV is defined in a similar manner as the proportion of the number of true regions in the detected clusters.
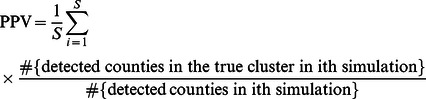
(7)


We can also weight each region with its population, and hence obtain population based SEN and PPV.
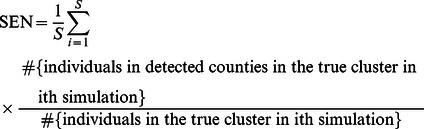
(8)

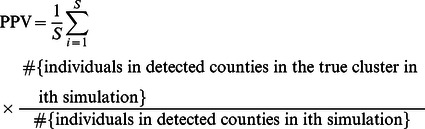
(9)


For the SEN and PPV, the larger the better they are, with 100% being the optimal. Here, the detected clusters are defined as follows: a critical value corresponding to a 0.05 significance level was computed by identifying the 5000th highest maximum index from among the 99,999 random data sets generated under the null model. For each kind of hot spot cluster, all windows with index exceeding the critical value are candidate clusters. The window with the maximum index is reported as the most likely cluster. Then, we eliminate the remaining candidate clusters that overlap with most likely cluster, and report the one having the largest index as the second cluster. We then repeated this procedure for the third and the fourth clusters and so on. All reported clusters are detected clusters.

#### Simulation results

We obtain the estimate of SEN and PPV for the test statistics based on the Bernoulli, the Poisson and the Hypergeometric models, respectively, and find that the results of the two test statistics proposed by Kulldorff are exactly the same except that a few values differ slightly. Thus, we take the Poisson model as an example for Kulldorff’s methods (Table 1). There are several common features between Hypergeometric and Poisson models: 1) In general, the two test statistics perform very similarly; 2) The SEN decreases as the number/size of hot spot clusters increases; 3) The PPV does not show the 2nd feature and always maintains a high level; 4) In general, the SEN based on population is greater than that based on counties, likewise for the PPV.

**Table 1:** SEN and PPV of test statistics based on the Poisson and the Hypergeometric models.

				county		population	
				SEN (%)	PPV (%)	SEN (%)	PPV (%)
rural	◊	1	H	99.19	97.30	99.19	97.08
			P	99.81	97.73	99.81	97.37
	◊	2	H	87.37	96.11	95.98	95.93
			P	87.58	96.58	96.36	96.37
	◊	4	H	93.38	82.36	93.86	87.00
			P	93.49	83.23	93.97	87.87
		8	H	86.71	83.84	88.87	85.88
			P	86.70	85.12	88.94	87.18
		16	H	82.74	88.02	84.80	86.17
			P	82.20	88.89	84.35	87.17
urban	◊	1	H	90.22	80.96	90.22	83.19
			P	91.97	83.85	91.97	86.11
		2	H	86.70	81.36	88.09	81.94
			P	86.20	82.53	87.79	83.23
	*	4	H	86.09	76.66	86.21	79.83
			P	84.00	76.39	84.08	79.30
	*	8	H	83.47	73.48	86.40	81.26
			P	81.07	72.69	84.00	80.03
	*	16	H	82.71	73.49	85.78	84.53
			P	80.48	72.47	83.55	83.02
rural and mixed	◊	1	H	94.10	74.24	89.32	87.54
			P	94.70	75.46	89.77	88.32
	◊	2	H	84.22	77.69	88.43	89.61
			P	84.23	78.88	88.47	90.44
		4	H	84.27	71.79	84.50	87.53
			P	84.22	72.93	84.08	88.29
		8	H	78.31	77.39	82.21	89.10
			P	77.87	78.57	81.57	89.94
		16	H	74.12	84.43	80.22	90.21
			P	73.27	85.29	79.20	90.88
mixed and urban	◊	1	H	84.63	71.37	84.54	88.29
			P	84.94	73.37	84.86	89.56
		2	H	78.43	75.57	81.70	89.37
			P	78.32	77.04	81.64	90.50
		4	H	70.80	73.12	72.37	88.02
			P	68.80	73.97	69.80	88.34
		8	H	62.29	75.54	67.72	87.88
			P	59.69	75.66	64.14	87.67
	*	16	H	53.75	78.60	58.20	88.52
			P	50.83	78.32	54.21	87.69

P: Denote the test statistic based on the Poisson probability model.

H: Denote the test statistic based on the Hypergeometric probability model.

◊: Denote that the test statistic based on the Poisson model performs better.

* : Denote that the test statistic based on the Hypergeometric model performs better.

The SEN and PPV represent two sides of a test statistic for cluster detection. If the SEN and PPV based on one probability model both are greater than the corresponding values based on the other model, the test statistic based on the former model outweighs the latter under that scenario. From Table 1 it appears that: 1) the Hypergeometric model outweighs the Poisson model when the hot-spot cluster is in urban area, whereas the Poisson model outweighs the Hypergeometric model when the hot spot cluster is in the rural area. In other words, with the densely populated hot-spot cluster, the Hypergeometric model performs better, otherwise the Poisson model performs better; 2) the Hypergeometric model outweighs the Poisson model when the size of hot-spot cluster is large, and otherwise the Poisson model performs better. In summary, with densely populated or largely sized hot-spot clusters, the Hypergeometric model performs better, otherwise the Poisson model performs better.

## Discussion

First, we would like to review the motivation of this study. Our initial test statistic was the P value, but it failed in the simulation study. There is a lot of literature relating to the role of the P value and its origin, in which a great part are concerned with what is evidence in statistics and the debate over Fisher’s test of significance and Neyman-Pearson’s hypothesis testing. That is beyond the scope of this paper, and plenty of literature has interestingly discussed those questions [44]–[50].

The key assumption to the two kinds of test statistics is that there is no positive spatial autocorrelation, which implies that pairs of observations taken nearby are more similar than those taken far apart. As summarized by Tango [1], the positive spatial autocorrelation will be a key issue in statistical modeling of spatial epidemiology. For instance, when spatial regression is performed to determine what covariates contribute to a higher risk for a disease under study, it is critical to adjust for the spatial autocorrelation in the data. Otherwise, the risk will be overestimated, with biased p-values that are too small, providing “statistically significant” results when none exist. However, for detecting disease clustering or disease clusters, we should not adjust for the spatial autocorrelation since we are interested in detecting clusters due to such autocorrelation and, if they are adjusted away, important clusters might go undetected. The test statistic based on the Hypergeometric model is more similar with the one based on Poisson model in terms of the data structure, both for case-population data.

As the simulation study shows, with the identical assumption, the two kinds of methods perform similarly. The SEN and PPV based on population usually are greater than that based on counties, indicating that the cluster detection methods often “capture” the hot spot areas of high population, which would benefit more overall. Furthermore, the test statistic based on the Hypergeometric model performs better when used with densely populated or largely sized hot spot clusters; otherwise Kulldorff’s test statistics perform better. Although it appears relatively small, given the scarce resources available to most local health departments, a greater improvement would reduce the cost to investigate potential disease outbreaks. In the application of JE in Sichuan province, the two kinds of methods identified the same cluster, which may greatly help the health department allocate relevant resources to these areas for JE prevention.

Further refinements of the new test statistic may include clusters that are not circular, but instead irregularly shaped ones. Space-time clusters extensions to the proposed method are also straightforward.
